# The Effect of Root Canal Preparation on the Development of Dentin Cracks

**Published:** 2012-10-13

**Authors:** Amin Salem Milani, Mohammad Froughreyhani, Saeed Rahimi, Mohammad Asghari Jafarabadi, Sara Paksefat

**Affiliations:** 1. Dental and periodontal disease research center, Dental School, Tabriz University of Medical Sciences, Tabriz, Iran.; 2. Department of Endodontics, Dental School, Tabriz University of Medical Sciences, Tabriz, Iran.; 3. Medical Education Research Center, Department of Statistics and Epidemiology, Faculty of Health, Tabriz University of Medical Sciences, Tabriz, Iran.

**Keywords:** Instrumentation, Protaper Rotary System, Root Canal Preparation, Tooth Fractures

## Abstract

**Introduction:**

Root fracture is not an instant phenomenon but a result of gradual development of tiny craze lines in tooth structure. Recent studies have shown that canal instrumentation has the potential to cause dentinal cracks. The purpose of this study was to evaluate and compare the formation of dentinal cracks caused by ProTaper rotary system to hand instrumentation.

**Materials and Methods:**

This in vitro study was carried out using 57 mandible incisor teeth. The teeth were decoronated. The roots were then examined to exclude cracked samples. A standard model for PDL simulation was used. The teeth were randomly divided into two experimental and one control group (n=19). The teeth in the experimental groups were prepared using hand or ProTaper Universal rotary instrumentation. The teeth in the control group were left unprepared. The teeth were then sectioned horizontally 3 and 6 mm from the apex, and the number of various dentinal defects was recorded using a dental operating microscope. The differences between groups were analyzed with Fisher’s exact test.

**Results:**

The hand group demonstrated significantly more defects than the control group (P=0.001). However, there was no significant difference between the rotary compared to the control and hand groups (P>0.05). There was no significant difference between groups with regards to fracture (P>0.05). Other defects including internal, external and surface cracks were more frequent in the hand than in the control or rotary groups (P=0.02), but the difference was not significant between the rotary and control groups (P>0.05).

**Conclusion:**

Canal preparation, whether hand or rotary, produces structural defects in dentin. The ProTaper rotary system when used according to the manufacturer’s instructions, tends to produce fewer cracks and can be considered a safe preparation technique.

## Introduction

Vertical root fracture (VRF) is a serious clinical problem often compromising the prognosis of teeth. Recent studies have shown that root fracture is not an instant event but rather gradual propagation of tiny, less pronounced craze lines in tooth structure [1][2]. Structural defects in a tooth have been shown to influence fracture strength [3] as stress is exponentially amplified at the tips of these cracks [4]. Endodontic procedures are generally blamed as the main cause of VRF [5][6][7]. The obturation stage has been believed to be the main culprit [7][8]. A fracture is a communicating crack; i.e. one that extends from the root canal space all the way to the outer root surface. External crazing or cracks includes cracks that extend from the root surface into dentin without reaching the canal lumen. Some recent studies have shown that canal instrumentation also has the potential to cause dentinal cracks [9][10][11]. Some studies have focused on various rotary instruments and showed a higher incidence of dentinal crack as a result of these techniques [9][10][11][12]. Shemesh et al. showed that canal preparation using a GT-file can produce dentinal cracks in mandibular premolars [9]. Similar results were achieved by Bier et al. when they prepared mandibular premolars using Profile and GT files [10]. An increasing number of Nickel-Titanium (NiTi) rotary systems have been introduced and despite various clinical advantages over hand instrumentation, rotary instruments can generate increased stress within the root canal [13] and additional rotation of the instruments within the canal is necessary to complete instrumentation with rotary files compared with hand ones [10][14]. These factors have been suggested to contribute to increased dentinal crack formation by rotary instrumentation [10][11].

ProTaper Universal (Dentsply, Maillefer, Ballaigues, Switzerland) is one of the commonly used NiTi rotary systems worldwide. Originally the ProTaper system comprised six instruments, three shaping and three finishing files [15]. The cross-section of the files demonstrates a modified k-file with sharp cutting edges and no radial lands. The unique design of this system is the varying taper along the files’ long axes. The taper increases coronally in the shaping files but the finishing files have the reverse pattern. The finishing files (F1, F2, and F3) have tip diameters of 0.2, 0.25, and 0.3 mm and apical tapers of 0.07, 0.08, and 0.09 mm, respectively [15]. The large apical tapers produced by this system can theoretically cause more dentinal cracks, especially in small weak roots [16]. The purpose of this study was to evaluate the ProTaper Universal rotary system and hand instrumentation regarding formation of dentinal cracks.

## Materials and Methods

This in vitro study was carried out using fifty-seven extracted mandibular incisors without root caries or root curvature. The length of the roots and the dimensions at the cementoenamel junction (CEJ) were measured using a gauge (Buffalo Dental Manufacturing C.D., Syosset, NY, USA). Teeth with 10-16 mm root length, 4-6 mm buccolingual diameter and 3-4 mm mesiodistal diameter were included in the study. The root surfaces were cleaned and the teeth were stored in 5.25% sodium hypochlorite (NaOCl) for an hour. The teeth were decoronated 3 mm coronal to the CEJ using an Isomet low-speed saw (Buehler Ltd, Evanston, IL, USA) with water cooling. The roots were examined with a dental operating microscope (DOM) (Carl Zeiss, Oberkochen, Germany) under 40 × magnification to exclude cracked samples. Canal length was measured by introducing a #15 K-File (Dentsply Maillefer, Switzerland) into the canal until the tip was visible at the apical foramen under 4 × magnification. One millimeter was subtracted from the canal length and recorded as the working length. Teeth with two canals, open apices or calcified canals were discarded. Fifty-seven teeth were deemed suitable for the study.

A standard model for PDL simulation was used with some modifications [17][18]. The teeth were placed in melted wax up to 1 mm below the CEJ. After cooling, the teeth were embedded in 2×2×2 cm blocks filled with gypsum (Moldano blue™, Heraues Kulzer, Hanau, Germany). After setting, the teeth were removed and the wax was cleaned from the root surface and “sockets” using warm water. The “sockets” were then filled with a polyether impression material (Impregum Soft, 3M ESPE, Seefeld, Germany) using a molding syringe. The teeth were then reinserted into the respective “sockets”. The excess impression material was removed with a scalpel. The teeth were randomly assigned to the following two experimental and one control groups. In group 1 (n=19), the canals were prepared with a stainless steel K-Flexofile (Dentsply Maillefer, Ballaigues, Switzerland) to a master apical file #30 with 0.5 mm increments step-back using Flexofiles #35 to 60. Each file was used to prepare 10 canals. The Flexofile #30 was used to recapitulate between each file size.

**Figure 1:**
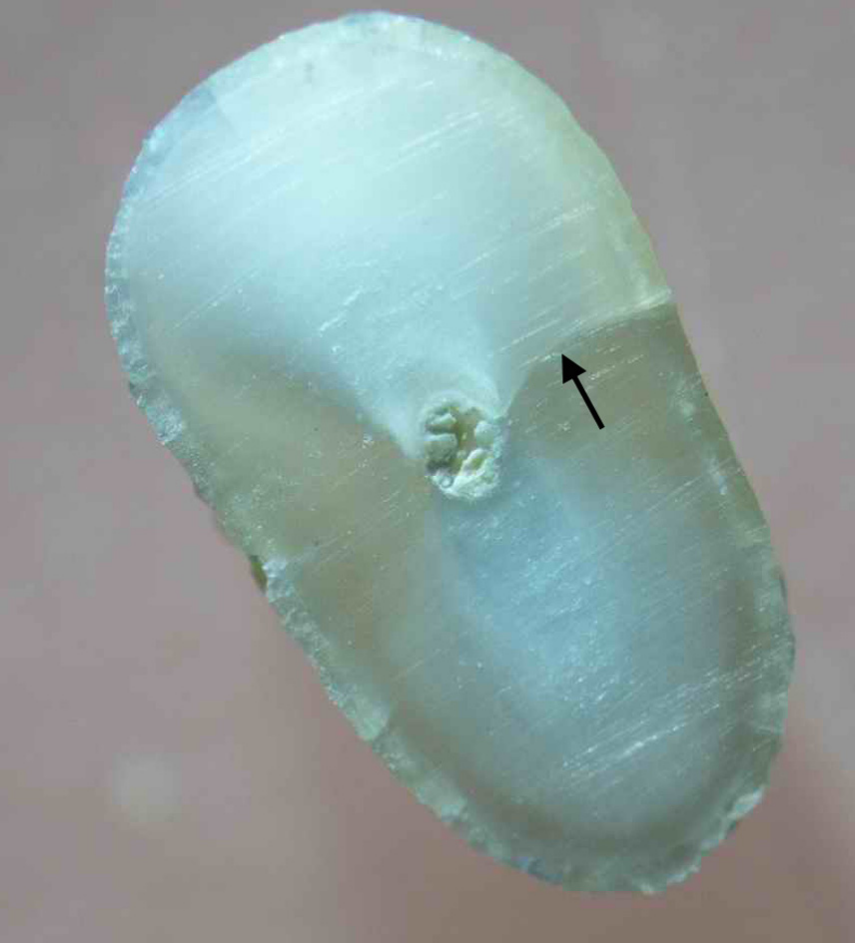
Black arrow pointing to a fracture

**Figure 2:**
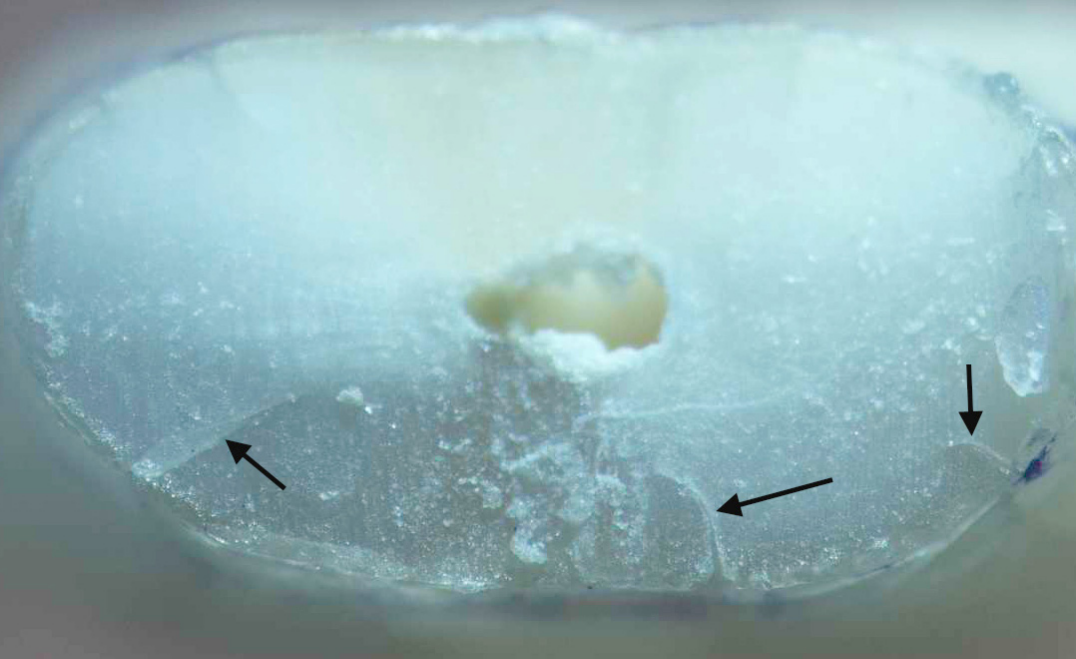
Black arrows pointing to external craze lines

In group 2 (n=19), ProTaper Universal rotary files (Dentsply Maillefer, Switzerland) were used in a 1:64 contra-angle handpiece (NSK, Nakanishi Inc, Japan) to prepare the canals. According to the manufacturer’s instructions, the Sx file was used to enlarge the coronal portion and then the S1, S2, F1, F2, and F3 files were used to full working length with a pecking motion applying gentle apical pressure. The files were removed and the dentinal debris cleaned off after three peckings until the files reached the working length. Each file was used to prepare 10 canals. The teeth in the control group were left unprepared. In all experimental groups, Diaprep (Diadent, Seoul, Korea) was used as lubricant and chelating agent during instrumentation. Each canal was irrigated with 1% NaOCl between each instrument using a syringe with a 27-guage needle. A total of 10 mL of NaOCl was used for each canal. Following instrumentation, the teeth were removed from the “sockets” and washed under running water. Each canal was irrigated with 0.5 mL of 17% EDTA solution (Ariadent, Tehran, Iran) for 30 seconds as the final rinse. The roots’ surface was then examined with a DOM under 40× magnification with illumination and the numbers of surface cracks were recorded. To make the examiner blind to the experimental groups, the roots were coded by random numbers corresponding to their group by another author. The samples were then sectioned horizontally at 3 and 6 mm from the apex with a low-speed saw under water cooling (Leica SP1600, Wetzlar, Germany). The slices were examined with DOM, and the number and type of dentinal defects were recorded and classified using Wilcox et al. classification [9][19] with some modification. “Fracture or communicating crack” was defined as any crack extending from the root canal space all the way to the outer root surface [19] (Figure 1). “Other defects” were other craze lines that did not extend from the root canal to the outer root surface. “Other defects” included internal and external craze lines. “Internal craze lines” included cracks extending from the canal wall into the dentin without reaching the root surface; however, “external craze lines” were defined as cracks extending from the root surface into dentin without reaching the canal lumen (Figure 2).

## Results

**Figure 3:**
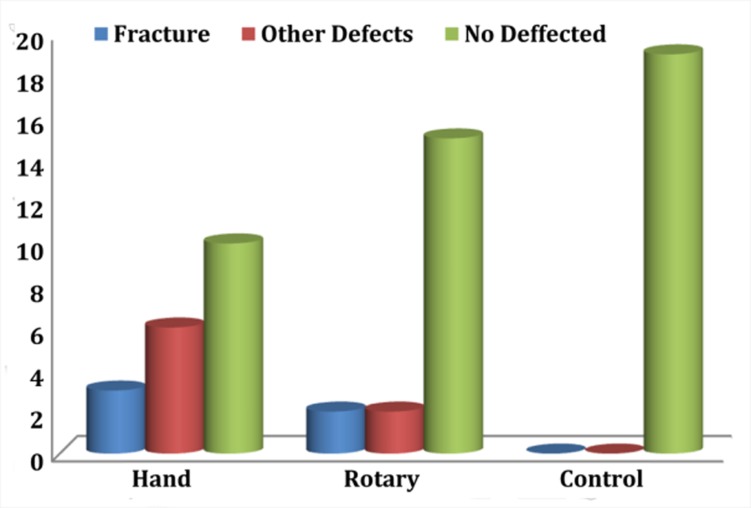
Number of different types of defects resulting from canal instrumentation

The results are summarized in Table 1.The unprepared control group showed no defects. Considering all defects, Fisher’s exact test showed statistically significant differences between groups (P=0.002). When considering all defects in all sections, the hand group demonstrated significantly more defects than the control group (P=0.001). However, there was no significant difference between the rotary-control and rotary-hand groups (P>0.05). Considering the fractures (communicating cracks) in all sections, there was no significant difference between groups (P=0.351) (Figure 1). When analyzing other defects in all sections, the hand group demonstrated significantly more defects than control or rotary groups (P=0.02), but the difference was not significant between the rotary and control groups (P>0.05) (Figure 3). The number of defects in 3- and 6-mm sections and surface cracks was not significantly different (P>0.05).

**Table 1:** The number of teeth with different types of defects as a result of instrumentation

**Groups (*n=*19)**	**Defected**				**Not defected**
	**Fractures**	**Internal craze lines**	**External craze lines**	**Surface cracks**	
**Hand**	3 (1, 2) a	0	4 (2, 2)	2	10
**Rotary**	2 (1, 1)	0	1 (1, 0)	1	15
**Control**	0	0	0	0	19

^a^ The numbers in parenthesis denote the number of teeth with cracks in 3- and 6-mm sections, respectively.

### Statistical Analysis

Fisher’s exact test was performed to compare the incidence of different crack types between groups using SPSS version 16 (SPSS Inc., Chicago, USA.). The level of significance was set at P=0.05.

## Discussion

Currently, NiTi rotary instruments are routinely used for canal preparation. A concern in some recent studies about rotary instrumentation is the formation of dentinal cracks that may eventually develop into complete fractures. There is some evidence that the design and taper of rotary files influence crack formation [9][10][12]. We used the ProTaper Universal rotary system in this study. According to the manufacturer’s instructions, finishing files of this system were used to prepare the apical portion of the canals. The large apical taper of finishing files in this system (up to 0.09 mm) generates increased stress on dentinal walls as compared to other rotary systems [10], which may increase the incidence of dentinal cracks. Furthermore, despite most rotary systems being used in a crown down manner, ProTaper Universal files are used with a “single length technique” [20]. Some studies have shown increased crack formation as a result of crown down rotary instrumentation [9][11][12]. But the effect of a “single length technique” rotary preparation on crack formation is still unclear.

Similar studies have used mandibular premolars [9][11][12]. We used mandibular incisors in our study, because these teeth are probably more prone to be influenced by forces during instrumentation as a result of their smaller dimensions and thin dentinal walls. Our hypothesis was that “if large tapered ProTaper files cannot induce cracks in weak mandibular incisors, it is unlikely that rotary files induce cracks in other teeth”.

In this study, the groups were matched by selecting similar-sized teeth and excluding samples with cracks or two canals. The sectioning procedure had no influence on crack formation because the control teeth did not show any defects. This is in agreement with other studies that used a similar method [10][11]. The apical taper of the last finishing file used was similar to the taper of roots prepared by hand instrumentation in this study. Therefore, there was a similarity between the two experimental groups with regards to the taper of the prepared roots, which increased the reliability of the outcome.

We used a 1:64 contra-angle handpiece to prepare the canals in the rotary group. Some studies comparing electric and air-driven handpieces with rotary nickel-titanium instruments have found no significant difference in file distortion or breakage between the two systems [20]. However, other studies have shown precise control of torque and speed by electric handpieces can affect the efficacy and durability of instruments [21][22]. Determining a file's rpm level is more difficult with an air handpiece than with an electric handpiece. For this reason it seems wise to use an electric handpiece with rotary files. Regardless of this controversy, there is an increasing use of rotary gear-reduction air-driven handpieces among dentists in Iran, possibly as a result of lower cost. Therefore, we used a 1:64 contra-angle. This makes our results more applicable to real clinical situations in our society.

The periodontal ligament (PDL), with its viscoelastic property, plays a major role in dissipating stress generated by load application to the teeth. Therefore, simulation of PDL is essential in studies that investigate the influence of forces on crack formation or fracture strength [17]. Some previous studies on the effect of instrumentation technique on crack formation did not consider this issue [9][10]. We used polyether impression material to simulate periodontal ligament as described by Bortoluzzi et al. [17]. Other studies have also used the same elastomeric material [23][24]. These elastomeric impression materials have nonlinear viscoelastic properties similar to PDL [25][26].

The results of the present study showed that canal instrumentation procedures produce dentinal cracks. This result is consistent with previous studies that demonstrated increased crack formation and fracture susceptibility of teeth as a result of instrumentation [9]. In our study, the number of communicating cracks-fractures was less than or equal to other defects in hand or rotary techniques, respectively (Table 1) which is also in agreement with previous studies [9][10]. Although fractures may be considered more important, we should not ignore the importance of other defects. They may propagate into complete fractures over time as a result of stresses produced during functional loadings or dental procedures.

In our study, no internal craze lines were observed and most of the defects were external cracks. These results are consistent with the results of a study by Shemesh et al. [9]. They demonstrated that many of the defects did not connect with canal space and were in places away from direct contact with instruments. One possible explanation is that the stress generated by instrumentation within the canal is transmitted to the outer surface of the tooth where it overcomes the bonds holding the dentin together [19]. In our study, the number of defects in the hand group was significantly more than in the control (P=0.020). However, there was no significant difference between the rotary and control groups (P=0.486) (Figure 3). This shows that hand instrumentation can produce dentinal defects. This seems in contrast with the study of Bier et al., which showed no influence of hand technique on crack formation. They used a balanced force technique in their study, while a step back technique was used in our study. Therefore, the technique of hand instrumentation may also have an effect on crack formation, which needs further research.

Some previous studies have shown increased dentinal defects as a result of rotary instrumentation compared with unprepared control teeth [9][10]. These studies did not use PDL simulation. In the present study, we used large tapered rotary files in mandibular incisors; however, by including PDL simulation, the relative number of defects in the rotary group was low and the difference was not significant. Sathorn et al. hypothesized that with rounder canal shapes and smoother canal surfaces as a result of rotary NiTi preparation [27][28][29] the fracture strength of teeth prepared with rotary systems would be more than of teeth instrumented with hand techniques [28]. It is worth mentioning that the relationship of increasing crack formation and fracture susceptibility needs further investigation.

## Conclusions

Within the limitations of this in vitro study, we can conclude that canal preparation produces more pronounced structural defects in dentin with the hand technique. The ProTaper Universal rotary system when used according to the manufacturer’s instructions tends to produce fewer cracks and is a relatively safe canal preparation technique.
